# Quantitative assessment of brain glymphatic imaging features using deep learning-based EPVS segmentation and DTI-ALPS analysis in Alzheimer’s disease

**DOI:** 10.3389/fnagi.2025.1621106

**Published:** 2025-07-16

**Authors:** Fenyang Chen, Tiantian Heng, Qi Feng, Rui Hua, Jiaojiao Wu, Feng Shi, Zhengluan Liao, Keyin Qiao, Zhiliang Zhang, Jianliang Miao

**Affiliations:** ^1^Department of Medical Imaging, Section One of Air Force Hangzhou Special Crew Sanatorium of PLA AIR Force, Hangzhou, China; ^2^Air Force Healthcare Center for Special Services, Hangzhou, China; ^3^Department of Radiology, Hangzhou First People’s Hospital Affiliated of Westlake University School of Medicine, Hangzhou, China; ^4^Department of Research and Development, Shanghai United Imaging Intelligence Co., Ltd., Shanghai, China; ^5^Department of Mental Health, Zhejiang Provincial People’s Hospital, Hangzhou, China; ^6^Department of Radiology, Zhejiang Hospital, Hangzhou, China

**Keywords:** Alzheimer’s disease, amnestic mild cognitive impairment, glymphatic system, V-shape bottleneck network, enlarged perivascular space, diffusion tensor imaging along perivascular spaces

## Abstract

**Background:**

This study aimed to quantitatively evaluate brain glymphatic imaging features in patients with Alzheimer’s disease (AD), amnestic mild cognitive impairment (aMCI), and normal controls (NC) by applying a deep learning-based method for the automated segmentation of enlarged perivascular space (EPVS) and diffusion tensor imaging analysis along perivascular spaces (DTI-ALPS) indices.

**Methods:**

A total of 89 patients with AD, 24 aMCI, and 32 NCs were included. EPVS were automatically segmented from T1WI and T2WI images using a VB-Net-based model. Quantitative metrics, including total EPVS volume, number, and regional volume fractions were extracted, and segmentation performance was evaluated using the Dice similarity coefficient. Bilateral ALPS indices were also calculated. Group comparisons were conducted for all imaging metrics, and correlations with cognitive scores were analyzed.

**Results:**

VB-Net segmentation model demonstrated high accuracy, with mean Dice coefficients exceeding 0.90. Compared to the NC group, both AD and aMCI groups exhibited significantly increased EPVS volume, number, along with reduced ALPS indices (all *P* < 0.05). Partial correlation analysis revealed strong associations between ALPS and EPVS metrics and cognitive performance. The combined imaging features showed good discriminative performance among diagnostic groups.

**Conclusion:**

The integration of deep learning-based EPVS segmentation and DTI-ALPS analysis enables multidimensional assessment of glymphatic system alterations, offering potential value for early diagnosis and translation in neurodegenerative diseases.

## 1 Introduction

Alzheimer’s disease (AD) is the most prevalent neurodegenerative disorder, characterized by a progressive decline in cognitive function and memory. With the global population aging, the burden of AD has emerged as an increasingly urgent public health challenge worldwide (Alzheimer’s Association, 2023). Its growing prevalence imposes substantial emotional and economic burdens on both families and healthcare systems alike (Bai et al., 2022). Amnestic mild cognitive impairment (aMCI) is widely recognized as an intermediate stage between normal aging and AD, associated with a high risk of progression to dementia, albeit with some potential for reversibility (Mian et al., 2024). Although several clinical strategies have been developed for the treatment of AD, their therapeutic efficacy remain limited and unsatisfactory (Passeri et al., 2022). Consequently, identifying key biological alterations during the aMCI stage is crucial for enabling early diagnosis and intervention (Porsteinsson et al., 2021).

The glymphatic system, a brain-wide perivascular network responsible for the clearance of metabolic waste, has attracted increasing attention for its potential role in the pathophysiology of AD (Rasmussen et al., 2018). Driven by arterial pulsations, it facilitates the exchange between cerebrospinal fluid (CSF) and interstitial fluid (ISF), thereby enhancing the clearance of neurotoxic proteins such as β-amyloid and tau (Liu et al., 2022) Aquaporin–4 (AQP4), a major water channel predominantly localized at the astrocytic endfeet, undergoes notable age-related changes, particularly a reduction in its perivascular polarization. This loss of perivascular polarization has been closely associated with AD-related pathological changes and may contribute to the brain’s heightened vulnerability to neurodegeneration in aging populations (Zeppenfeld et al., 2017). In animal models deficient in AQP4, the circadian regulation of glymphatic clearance is disrupted, leading to impaired elimination of brain metabolites and further supporting the involvement of glymphatic dysfunction in AD progression (Hablitz et al., 2020). Taken together, these findings highlight the necessity of a systematic evaluation of glymphatic function in AD and aMCI, which could not only deepen our understanding of disease mechanisms but also facilitate the identification of early imaging biomarkers with potential clinical relevance.

Magnetic resonance imaging (MRI) has enabled noninvasive in vivo evaluation of the glymphatic system (Bae et al., 2023; Taoka and Naganawa, 2020; Zhang et al., 2021; Kamagata et al., 2024). Perivascular spaces (PVS), as key anatomical conduits within the glymphatic system, have garnered growing interest for their potential utility as imaging biomarkers of impaired brain clearance. Enlarged perivascular spaces (EPVS), frequently observed in regions such as the basal ganglia (BG) and centrum semiovale (CSO), may indicate underlying glymphatic drainage dysfunction (Lynch et al., 2023). The volume, number, and spatial distribution of EPVS have been associated with aging, cognitive decline, and various neurological disorders. However, current clinical assessments of EPVS primarily rely on visual rating scales, which are inherently subjective and lack methodological standardization (Moses et al., 2023).

To improve the standardization and automation of EPVS assessment, several deep learning–based segmentation approaches have been proposed in recent years (Waymont et al., 2024). Among these methods, VB-Net—a modified version of V-Net incorporates bottleneck layers—has demonstrated superior performance in brain structure segmentation, due to its use of skip connections and ability to extract multi-scale features. This model achieved an average Dice similarity coefficient (DSC) greater than 0.90 for various small vessel disease markers in prior work, with high recall (0.953) and precision (0.923) rates for EPVS segmentation, validated against manual annotations by experienced radiologists (Zhu et al., 2022). These characteristics make it particularly well-suited for the detection of EPVS, which are often small in size, morphologically heterogeneous, and poorly delineated at their boundaries.

Among emerging techniques, diffusion tensor image analysis along perivascular spaces (DTI-ALPS) quantifies water diffusivity perpendicular to major white matter fiber tracts, offering a reproducible and noninvasive metric for assessing glymphatic function integrity (Taoka et al., 2017; Liu et al., 2024). This approach has been increasingly applied to assess glymphatic activity across a spectrum of neurological conditions, including neurodegenerative disorders, ischemic stroke, and cerebral small vessel disease (Hsu et al., 2023; Qin et al., 2023; Hong et al., 2024). Integrating automated EPVS quantification with DTI-ALPS analysis represents a promising strategy for the multidimensional evaluation of glymphatic function in individuals with AD and aMCI.

In this study, we aimed to implement a fully automated MRI-based EPVS segmentation framework using the VB-Net model, in combination with DTI-ALPS indices, to enable a comprehensive structural and functional evaluation of the glymphatic system in patients with AD and aMCI. We hypothesize that individuals with AD or aMCI exhibit varying degrees of glymphatic dysfunction, which can be detected through distinct imaging biomarkers. Furthermore, EPVS-derived metrics and DTI-ALPS indices may offer complementary insights, potentially improving disease detection and subtype differentiation.

## 2 Materials and methods

### 2.1 Participants and clinical information

AD and aMCI subjects were recruited in this cross-sectional study conducted at the memory clinic of Zhejiang Provincial People’s Hospital (Hangzhou, China) from September 2016 to March 2019. The normal control (NC) subjects were volunteers recruited at the Hospital Health Promotion Center. All participants were right-handed and signed an informed consent. This study was carried out in accordance with the Declaration of Helsinki, and all procedures were approved by the local ethics committee of Zhejiang Provincial People’s Hospital (No. 2012KY002).

The medical history, neuropsychological test, physical examination, laboratory inspection, and craniocerebral MRI scan data of all subjects were collected. The neuropsychological scales involved the mini-mental state examination (MMSE) and Montreal cognitive assessment (MoCA). The inclusion criteria for AD patients were revised NINCDS-ADRDA criteria with MMSE score ≤ 24 and MoCA score ≤ 26; higher scores indicated better cognition (Dubois et al., 2007). The inclusion criteria for aMCI patients were as follows: (1) complaints of memory impairment by patient, family, or physician; (2) normal clinical manifestations including cognitive function; (3) MMSE score > 24 and ≤ 27. The inclusion criteria for NC subjects were as follows: (1) no stroke, epilepsy, depression, or other neurological or mental diseases; (2) no hearing or visual impairment; (3) conventional craniocerebral MRI showed no infarction, hemorrhage, tumor, or other lesions; (4) MMSE score ≥ 28. The exclusion criteria for all three groups were: (1) stroke and infarction; (2) traumatic brain injury; (3) epilepsy, Parkinson’s disease, brain tumor, and other neurological diseases that led to memory impairment; (4) vascular dementia or mixed dementia; (5) severe anemia, hypertension, diabetes, and use of psychotropic drugs. Finally, 89 AD patients, 24 aMCI patients, and 32 NC were enrolled in this study.

### 2.2 MR image acquisition

The MRI data were acquired on a clinical MR scanner using an eight–element receiving coil (Discovery MR750 3.0T; GE Healthcare, Wisconsin, United States). The MRI protocols included T1-weighted imaging (T1WI), T2WI, T2-fluid attenuated inversion recovery (FLAIR), and diffusion-weighted imaging (DWI, b = 0 and 1,000) to exclude subjects with craniocerebral disease. The DTI data were acquired using gradient echo single-shot EPI sequence with 25 diffusion gradient square [echo time (TE) = 63.8 ms, repetition time (TR) = 8,612 ms, matrix = 256 × 256, field of view (FOV) = 192 × 192 mm^2^, slice thickness / slice spacing = 1.5/0 mm, 81 axial slices, voxel = 0.75 × 0.75 × 1.5 mm^3^, and b values 0 and 1,000 s/mm^2^]. Also, high-resolution three-dimensional (3D) T1-weighted magnetization-prepared fast gradient echo (MPRAGE) vector images were acquired (TE = 2.9 ms, TR = 6.7 ms, matrix = 256 × 256 mm^2^, FOV = 256 × 256 mm^2^, slice thickness/slice spacing = 1/0 mm, 192 sagittal slices, and voxel = 1 × 1 × 1 mm^3^).

### 2.3 EPVS preprocessing

Enlarged perivascular spaces are fluid-filled spaces surrounding small penetrating vessels, appearing as linear or ovoid structures typically < 3 mm in diameter. According to the STRIVE-2 criteria, EPVS are isointense to cerebrospinal fluid on all MRI sequences and lack a hyperintense rim on FLAIR. They are most commonly observed in the basal ganglia, centrum semiovale, and midbrain (Duering et al., 2023).

Image processing was conducted on the uAI Research Platform (uRP) (Wu et al., 2023), which integrates a generalized segmentation framework based on the VB-Net architecture, enabling robust extraction of various regions of interest (ROIs) (Zhu et al., 2022). In this study, VB-Net was employed to achieve efficient and reproducible automated segmentation of EPVS. The preprocessing steps for T1- and T2-weighted images were as follows: (a) N4 bias field correction was applied to the T1-weighted images to address magnetic field inhomogeneity; (b) a 3D VB-Net model deployed on the uRP platform was used to perform skull stripping on the T1-weighted images; (c) the Advanced Normalization Tools (ANTs) framework was used to co-register T1 and T2 images, and the brain mask from T1 was mapped onto the T2 images to enable skull removal in T2-weighted images; (d) both T1 and T2 images were resampled to an isotropic voxel size of 1 mm × 1 mm × 1 mm; (e) image intensities were normalized to a fixed range of [−1, 1].

**FIGURE 1:**
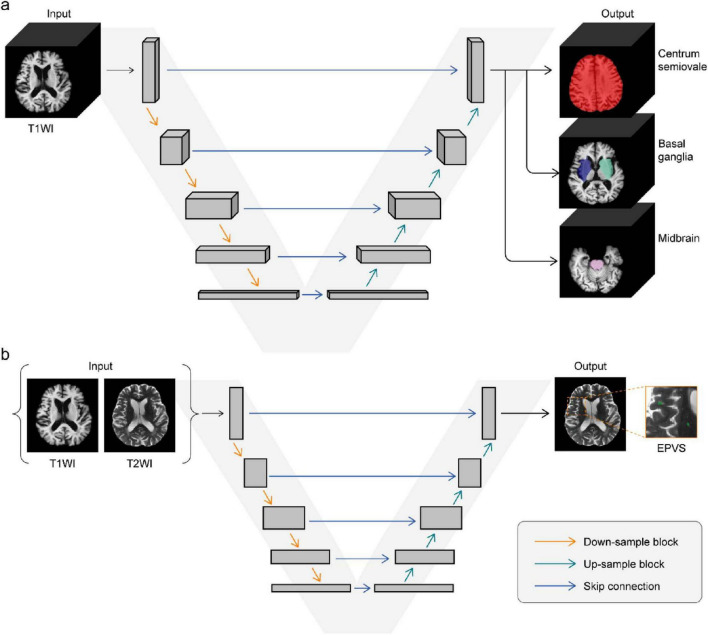
VB-Net employed for EPVS segmentation. **(a)** T1-weighted images were used as an input for a 3D VB-Net model to obtain segmentation of centrum semiovale, basal ganglia, and midbrain. **(b)** T1 and T2-weighted images were used as input for a 2D VB-Net model to obtain PVS segmentation.

Subsequently, the preprocessed T1–weighted images were input into a 3D VB-Net model to segment the CSO, BG, and midbrain regions, which served as anatomical references for localizing EPVS lesions. The 3D VB-Net model, based on a V-shaped bottleneck architecture, comprised input blocks, down-sampling / up-sampling blocks with residual structures, and output blocks. Bottleneck layers within each block reduced feature map dimensions to optimize GPU memory while preserving spatial details, and skip connections at each resolution fused high-level contextual features with fine-grained local information, enhancing boundary detection for heterogeneous regions like the BG and CSO. Trained on T1-weighted images from 1,800 subjects and tested on 295 subjects, the 3D VB-Net model achieved an average DSC of 0.92 and has been integrated into the uRP platform for end-to-end workflows via a graphical interface (Duering et al., 2023). Additionally, the key implementation files are hosted on GitHub^1^. For detailed PVS segmentation, the preprocessed T1 and T2 images were input into a 2D VB-Net model. The 2D VB-Net model leveraged a V-shaped bottleneck architecture with skip connections and multi-scale feature extraction, which enabled robust segmentation of EPVS despite boundary blurring and morphological heterogeneity. Bottleneck layers compressed feature maps to retain discriminative spatial details, while residual connections integrated low-level boundary information with high-level semantic features. To reduce false positives, segmented structures with lengths less than 2 mm were excluded. The remaining structures were retained and identified as EPVS lesions (as illustrated in Figure 1).

To evaluate the segmentation performance of the VB-Net model, the Dice similarity coefficient (DSC) was used to quantify the overlap between the AI-generated segmentation and the manually annotated ground truth. In this study, the model-generated EPVS results were reviewed and refined by two experienced radiologists (Rater 1: Jianliang Miao; Rater 2: Qi Feng), with these expert-corrected segmentations serving as the definitive ground truth. The raters were blinded to group diagnosis (AD, aMCI, NC) during their review to minimize bias. Consistency was assessed between the AI segmentation and each individual rater, as well as with the union and intersection of the two raters’ annotations. The DSC was calculated using the following formula:


$$\mathrm{DSC}=\frac{2\times \mathrm{TP}}{2\times \mathrm{TP}++\mathrm{FP}+\mathrm{FN}}$$

In this context, true positives (TP) refer to correctly identified EPVS voxels, false positives (FP) to non-EPVS voxels incorrectly labeled as EPVS, and false negatives (FN) to EPVS voxels that were missed. A higher DSC indicates better agreement between the AI-generated segmentation and the ground truth. In addition to DSC, we also calculated precision and recall to further assess segmentation performance. The formulas are as follows:


$$\mathrm{recall}=\frac{T\,P}{T\,P+F\,N}\,\mathrm{precision}=\frac{\mathrm{TP}}{\mathrm{TP}+\mathrm{FP}}$$

Based on the segmentation results, the total volume and number of EPVS lesions were extracted. In addition, EPVS volume fractions within the basal ganglia (BG) and centrum semiovale (CSO) regions were calculated for subsequent group comparisons and correlation analyses.

### 2.4 DTI image preprocessing

Diffusion-weighted images were corrected for Eddy current distortions and gradient direction using FSL 6.0 (Jenkinson et al., 2012). High-resolution 3D T1WI and DTI brain images extraction were captured using CAT12 toolbox (Gaser et al., 2024). Whole-brain volume of each subject was calculated using CAT12 for subsequent statistical analyses. The diffusion tensor model was then applied at each voxel using the DTIfit tool in FSL, and parameter values such as fractional anisotropy (FA) and mean diffusivity (MD) were calculated. Subsequently, FA maps were generated.

Furthermore, the linear registration tool in FSL was first used to align the 3D T1-weighted image to the individual DTI space, ensuring spatial consistency. Subsequently, the DTI images were registered to the MNI standard template using both linear and nonlinear registration, and the resulting spatial transformation matrices were obtained.

### 2.5 ALPS processing

After preprocessing, diffusion tensors were calculated in the x (left–right), y (anterior–posterior), and z (superior–inferior) directions at each voxel using FSL. Spherical region of interest (ROI) with 5-mm diameter was then placed on the MNI T1 template (MNI152_T1_1mm_brain, version 2.4) (Fonov et al., 2011; Figure 1). The center coordinates of the left and right ROIs in projection fibers were (24, −12, 24) and (−28, −12, 24), respectively, while those in association fibers were (36, −12, 24) and (−40, −12, 24) (Zhang et al., 2021). The ROI masks were inversely transformed to each subject’s T1 space using the deformation fields obtained from the prior registration. Manual verification was performed in FSLEYES to confirm the registration accuracy and ROI placement for each subject (Rater 1: Jianliang Miao; Rater 2: Qi Feng). Finally, the ALPS index was calculated based on the FA map using the following formula:


ALPS⁢index=(Dx⁢_⁢proj+Dx⁢_⁢assoc)/(Dy⁢_⁢proj+Dz⁢_⁢assoc)

**FIGURE 2:**
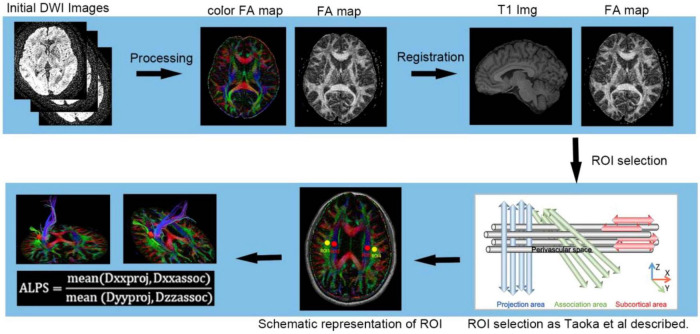
Process of selecting ALPS ROIs on the individual. The MNI coordinates of the centers of the left and right ROIs were (36, −12, 24) and (−40, −12, 24) in association fibers. The MNI coordinates of the centers of the left and right ROIs were (24, −12, 24) and (- @28, - 12, 24) in projection fibers. The corresponding transformation matrix produced in the registration Process is utilized for the inverse registration of the ROI in MNI space to each individual’s FA map, which is subsequently subjected to clinical expert review to facilitate precise results.

The ALPS index was calculated separately for the left and right hemispheres, and their mean was used as the individual-level measure of overall glymphatic function (Toh and Siow, 2021), as illustrated in Figure 2.

### 2.6 Statistical analysis

Statistical analyses were performed using SPSS version 26.0 and FSL, with statistical significance defined as *P* < 0.05. Demographic and neuropsychological variables were analyzed using SPSS. The normality of all variables was assessed using the Shapiro–Wilk test. Sex differences were assessed using the chi-square test. For group comparisons of ALPS indices and PVS-related metrics, either analysis of covariance (ANCOVA) or the Kruskal–Wallis H test was applied, depending on the data distribution. When significant differences were observed, post hoc comparisons were corrected using either the Bonferroni or Dunn method as appropriate.

Partial correlation analyses were conducted to examine associations between ALPS index, EPVS volume, number, and volume fraction with cognitive test scores, controlling for age, sex, years of education, and total brain volume as covariates. Additionally, correlations between ALPS indices and EPVS metrics were assessed to investigate the potential interrelationship between these two glymphatic-related indicators in the disease context. A *P* < 0.05 was considered statistically significant for all analyses.

## 3 Results

### 3.1 Demographics and clinical information

A total of 89 patients with AD, 24 individuals with aMCI, and 32 cognitively normal controls (NC) were included in the study. No significant differences were found among the three groups in terms of age, sex, or years of education (*P* > 0.05 for all; see Table 1). In contrast, both MMSE and MoCA scores differed significantly across groups (*P* < 0.05 for both).

**TABLE 1:** Demographics and clinical characteristics.

Characteristic	AD (*n* = 89)	aMCI (*n* = 24)	NC (*n* = 32)	Statistic	*P*-value
Age (mean ± SD)	68.750 ± 9.292	67.250 ± 9.317	65.410 ± 7.551	3.626	0.163^a^
Sex (male/female)	40:49	12:12	12:20	3.781	0.151^b^
Education (years)	7.830 ± 4.360	8.250 ± 4.173	8.720 ± 2.543	1.611	0.447^a^
MMSE, mean (SD)	17.270 ± 5.670	25.920 ± 0.974	28.590 ± 0.798	108.528	<0.001*^a^
MOCA, mean (SD)	13.38 ± 6.173	20.880 ± 3.982	27.280 ± 1.420	91.082	<0.001*^a^

AD, Alzheimer’s disease; aMCI, Amnestic mild cognitive impairment; NC, normal control; MMSE, Mini-Mental State Examination; MOCA, Montreal Cognitive Assessment. ^a^: Kruskal–Wallis H test;^b^: chi-square test; *P-value < 0.05.

### 3.2 EPVS results

**FIGURE 3:**
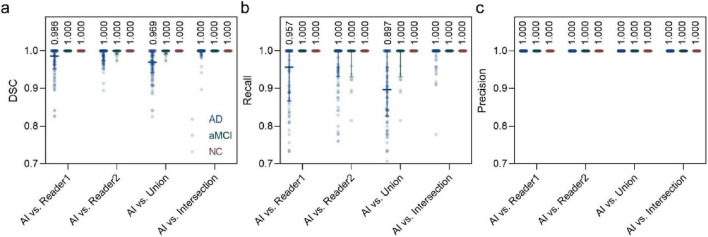
Scatter plots showing the performance of VB-Net in segmenting EPVS lesions. **(a)** DSC, **(b)** recall, and **(c)** precision were calculated from the VB-Net segmented results and ground truths (i.e., Reader1, Reader2, union of Readers, and intersection of Readers). The numbers annotated in the plot represent the median. Each subgroup was also labeled with the median and its interquartile range.

The segmentation performance of the VB-Net model demonstrated high concordance with manual annotations in terms of DSC, recall, and precision (see Figure 3). Specifically, the median DSC between VB-Net and Rater 1 was 0.969 (recall: 0.960; precision: 1.000), while that between VB-Net and Rater 2 was 0.986 (recall: 0.954; precision: 1.000) (see Table 2). The inter-rater agreement between the two experts was also high, with a median DSC of 0.996 (Figure 3).

**TABLE 2:** Quantitative performance of VB-Net in segmenting EPVS lesions.

	AI vs. reader1	AI vs. reader2	AI vs. union	AI vs. intersection
DSC	0.969 (0.960, 1.000)	0.986 (0.956, 1.000)	0.961 (0.956, 1.000)	0.996 (0.960, 1.000)
Recall	0.960 (0.919, 1.000)	0.954 (0.934, 1.000)	0.934 (0.887, 1.000)	0.990 (0.960, 1.000)
Precision	1.000 (0.960, 1.000)	1.000 (0.960, 1.000)	1.000 (0.960, 1.000)	1.000 (0.960, 1.000)

All numbers were calculated from the overall participants. Each metric was represented with a median (25% Percentile, 75% Percentile).

Based on the segmentation results, the total volume and number of EPVS lesions were quantified, along with their volume fractions in the basal ganglia (BG) and centrum semiovale (CSO) regions. As none of these variables met the assumption of normality, group comparisons were conducted using the Kruskal–Wallis H test. Significant group differences were identified in total EPVS volume, lesion count, and BG volume fraction (all *P* < 0.01; see Table 3). Post hoc comparisons with Bonferroni correction revealed that differences between the AD and aMCI groups, as well as between the AD and NC groups, remained statistically significant (all *P* < 0.05).

**TABLE 3:** Total volume and number of EPVS lesions segmented from the VB-Net.

	AD	aMCI	NC	*P*	*P*_(1__vs. 2)_	*P*_(1__vs. 3)_	P_(2__vs. 3)_
**All EPVS lesions**							
1. Volume	457.5 (282.4, 735.4)	330.9 (192.6, 512.4)	320.6 (222.3, 473.7)	0.003	0.030	0.014	> 0.999
2. Number	19.0 (11.0, 29.0)	14.0 (8.5, 20.5)	12.0 (10.0, 17.5)	0.003	0.036	0.012	> 0.999

Each metric was represented with a median (25% percentile, 75% percentile). Comparison analyses were performed by Kruskal–Wallis H test, followed by Dunn’s multiple comparisons tests.

### 3.3 Correlations between EPVS metrics and clinical variables

**FIGURE 4:**
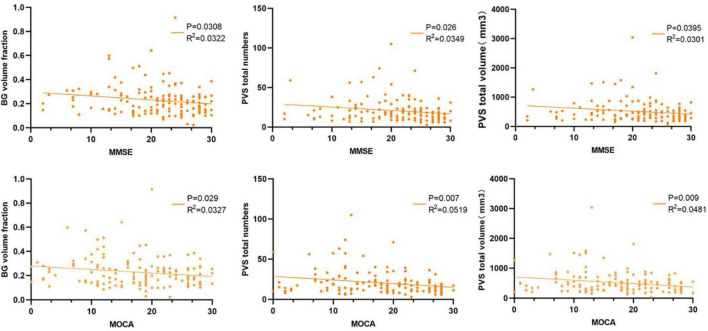
Correlation analyses between the BG volume fraction, total EPVS volume, and EPVS number with MMSE, MOCA scales.

Total EPVS volume, lesion number, and BG volume fraction were all significantly negatively correlated with MMSE (*R*^2^ = 0.030, *P* = 0.040; *R*^2^ = 0.035, *P* = 0.026; *R*^2^ = 0.032, *P* = 0.031) and MoCA (*R*^2^ = 0.048, *P* = 0.009; *R*^2^ = 0.052; *P* = 0.007; *R*^2^ = 0.033, *P* = 0.029) scores (see Figure 4), indicating that greater EPVS volume and number—whether in volume, number, or BG distribution—is associated with poorer cognitive performance.

### 3.4 ALPS results

As the ALPS index followed a normal distribution, analysis of covariance (ANCOVA) was used to compare group differences. Significant differences were observed among the three groups in the left ALPS index (*F* = 28.893, *P* < 0.001), right ALPS index (*F* = 3.595, *P* = 0.030), and mean ALPS index (*F* = 36.643, *P* < 0.001). After Bonferroni correction, both the left and mean ALPS indices remained significantly different across all pairwise group comparisons—AD *vs*. aMCI, aMCI *vs*. NC, and AD *vs*. NC (left ALPS: *P* < 0.001, *P* = 0.003, *P* = 0.002; mean ALPS: *P* < 0.001, *P* < 0.001, *P* = 0.005). For the right ALPS index, significant differences were found between the AD and aMCI groups, as well as between the AD and NC groups (*P* < 0.001, *P* = 0.002, *P* = 0.0227; see Table 4).

**TABLE 4:** Results of the EPVS and ALPS index in AD, aMCI, and NC participants.

	AD (*n* = 89)	aMCI (*n* = 24)	NC (*n* = 32)	*P*-value^c^	*P*-value^d^
EPVS total volume	457.5 (282.4, 735.4)	330.9 (192.6, 512.4)	320.6 (222.3, 473.7)	0.003*^b^	0.010*, 0.050*, 0.912
EPVS total number	19.000 (11.000, 29.000)	14.000 (8.500, 20.500)	12.0 (10.000, 17.500)	0.003*^b^	0.009*, 0.059, 0.901
BG EPVS volume fraction	0.236 (0.149, 0.311)	0.184 (0.110, 0.232)	0.172 (0.134, 0.240)	0.005*^b^	0.034*, 0.027*, 0.950
CSO EPVS volume fraction	0.000 (0.000, 0.014)	0.000 (0.000, 0.017)	0.000 (0.000, 0.008)	0.729^b^	
ALPS index-L	1.312 ± 0.016	1.429 ± 0.047	1.576 ± 0.031	<0.001*^a^	< 0.001*, 0.003*, 0.002*
ALPS index-R	1.303 ± 0.018	1.472 ± 0.043	1.565 ± 0.031	0.030*^b^	<0.001*, 0.002*, 0.227
ALPS index	1.308 ± 0.015	1.450 ± 0.040	1.570 ± 0.027	<0.001*^a^	< 0.001*, <0.001*, 0.005*

AD, Alzheimer’s disease; aMCI, Amnestic mild cognitive impairment; NC, normal control; EPVS volume fraction = EPVS volume/whole brain volume; BG, basal ganglia; CSO, centrum semiovale. ALPS index: average of ALPS index-L and -R. *P-value < 0.05. ^a^: ANOVA test. ^b^: Kruskal–Wallis H test. ^c^: *P*-value for comparison among AD, aMCI, and NC by ANOVA or Kruskal–Wallis H test.^d^: *P*-value for Bonferroni’s correction between AD-NC, AD-aMCI, and aMCI-NC .

### 3.5 Correlations between alps index and clinical variables

**FIGURE 5:**
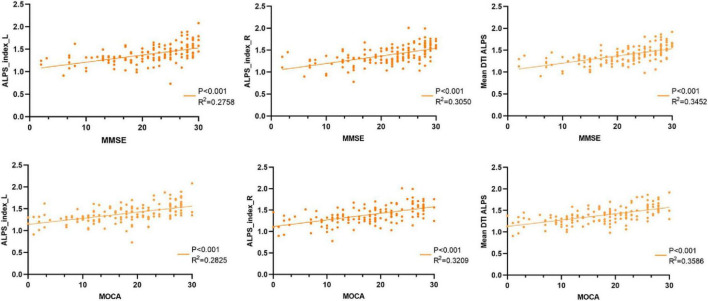
Correlation analyses between the ALPS index and MMSE, MOCA scales.

Partial correlation analysis revealed significant positive associations between ALPS indices (left, right, and mean) and both MMSE and MoCA scores (*P* < 0.001 for all; see Figure 5). Specifically, the left ALPS index correlated with MMSE (*R*^2^ = 0.2758, *P* < 0.001) and MoCA (*R*^2^ = 0.2825, *P* < 0.001), and the right ALPS index with MMSE (*R*^2^ = 0.3050, *P* < 0.001) and MoCA (*R*^2^ = 0.3209, *P* < 0.001). The mean ALPS index showed the strongest associations with MMSE (*R*^2^ = 0.3452, *P* < 0.001) and MoCA (*R*^2^ = 0.3586, *P* < 0.001).

### 3.6 Correlation between EPVS metrics and ALPS index

To further investigate the relationship between EPVS and the ALPS index, Spearman correlation analysis was performed. The results revealed significant negative correlations between the mean ALPS index and total EPVS volume, lesion number, as well as EPVS volume fractions in the BG and CSO regions (*P* = 0.022, *P* = 0.037, *P* = 0.029, *P* = 0.011, respectively), suggesting a potential link between structural EPVS volume and number and functional glymphatic impairment.

## 4 Discussion

This study systematically evaluated the structural and functional integrity of the glymphatic system in patients with Alzheimer’s disease (AD) and amnestic mild cognitive impairment (aMCI) using diffusion tensor image analysis along perivascular spaces (DTI-ALPS) and automated EPVS metrics derived from deep learning segmentation. The results demonstrated that ALPS indices were significantly reduced in both AD and aMCI groups compared with normal controls, and were positively correlated with cognitive scores, suggesting that glymphatic dysfunction may emerge as early as the aMCI stage. Using a deep learning–based VB-Net model, we achieved high-accuracy automatic segmentation of EPVS lesions, substantially improving the efficiency and reproducibility of structural quantification. Further analysis revealed significantly increased total EPVS volume and basal ganglia (BG) volume fraction in patients with AD and aMCI, both of which were closely associated with cognitive decline. These findings reflect underlying impairment in brain waste clearance and support the potential of EPVS as an early neuroimaging biomarker of neurodegeneration. Importantly, a significant negative correlation was also observed between ALPS indices and EPVS metrics.

The glymphatic system plays a critical role in clearing brain metabolic waste through CSF–interstitial fluid exchange driven by arterial pulsation, and has been increasingly implicated in the pathogenesis of various neurodegenerative disorders (Rasmussen et al., 2018). Impairments such as AQP4 depolarization, blood–brain barrier disruption, and restricted interstitial flow may compromise glymphatic clearance efficiency, thereby facilitating the accumulation of toxic proteins like β-amyloid (Aβ) and tau in the brain parenchyma (Harrison et al., 2020; Wang et al., 2023). In our study, the ALPS index was significantly reduced in the aMCI group and showed a strong association with cognitive decline, suggesting that glymphatic dysfunction may precede overt clinical symptoms of dementia and serve as a potential early imaging biomarker. This finding is consistent with previous work by Huang et al. (2024). Notably, although ALPS indices were reduced bilaterally, the left hemisphere exhibited a more pronounced decline, consistent with the left-lateralized aging pattern reported by Taoka et al. in healthy older adults (Taoka et al., 2017). However, other studies have reported right-hemispheric vulnerability (Bao et al., 2025), which may be attributable to differences in ROI placement, imaging resolution, or cohort characteristics, highlighting the need for further validation using larger samples and standardized methodology.

As a key anatomical component of the glymphatic system, PVS facilitate CSF transport and clearance. The volume and number of EPVS has been linked to cognitive impairment in neurodegenerative diseases, and PVS dilation may reflect impaired glymphatic clearance, resulting in interstitial metabolite accumulation and subsequent structural expansion (Hilal et al., 2018; Jie et al., 2020). Due to the low signal intensity, small size, and morphological variability of EPVS, manual annotation remains time-consuming and inherently subjective. In this study, we employed a deep learning–based VB-Net algorithm (Duering et al., 2023) to achieve accurate EPVS segmentation on both T1WI and T2WI images. The model demonstrated excellent performance across imaging modalities, with a Dice coefficient exceeding 0.90. Owing to its lightweight architecture and high inference efficiency, VB-Net is well-suited for deployment in clinical settings.

By extending the analysis to include individuals with aMCI, we further compared regional EPVS metrics and found significantly elevated EPVS volume fractions in the BG in both AD and aMCI groups, whereas no significant differences were observed in the CSO. This finding aligns with recent longitudinal studies reporting faster EPVS expansion in the BG than in the CSO, with the BG region exhibiting greater sensitivity to Aβ and tau pathology (*B* = 0.05 vs. 0.03) (Menze et al., 2024). Another study involving Aβ-PET–positive AD patients also reported increased BG-EPVS volume and number in late-onset AD, which was associated with hypertension and lacunar infarction, whereas CSO-EPVS did not exhibit similar associations and may instead reflect cerebral amyloid angiopathy (CAA)–related changes (Jung et al., 2024). Our findings support the notion that BG-EPVS may serve as a regional marker for early glymphatic dysfunction. However, other studies have reported elevated CSO-EPVS volume and number in neurodegenerative conditions, with PVS dilation associated with CSF AQP4 and total tau levels, suggesting that CSO changes may also reflect glymphatic dysfunction and neuronal injury (Sacchi et al., 2023). Current inconsistencies in the regional sensitivity and pathophysiological significance of EPVS may stem from differences in ROI definitions, quantification methods, and cohort characteristics, underscoring the need for further standardized investigations.

Furthermore, we identified a significant negative correlation between ALPS indices and EPVS metrics, suggesting that structural EPVS dilation may be accompanied by functional glymphatic impairment, consistent with a structure–function association in glymphatic disruption.

Despite the strengths of our study, several limitations should be acknowledged. First, the sample size was relatively small and exhibited group imbalance, and larger cohorts will be necessary to enhance the generalizability of the findings. Second, the calculation of the ALPS index depends on image registration and ROI placement. Although a standardized protocol was employed, further studies are needed to validate its robustness and reproducibility across different scanners and processing pipelines. Future studies could incorporate advanced neuroimaging biomarkers such as amyloid and tau PET quantification to improve differentiation between AD and aMCI subtypes. Moreover, glymphatic dysfunction may be interact with alterations in metabolic and lipidomic pathways. Integrating multiomic features, including metabolites and lipids, may offer a promising approach to improve both mechanistic understanding and diagnostic prediction (Zhou, 2021).

## 5 Conclusion

In conclusion, this study integrated ALPS and EPVS metrics to demonstrate the presence of both functional and structural glymphatic abnormalities at the aMCI stage. We proposed a combined assessment framework for glymphatic function and structure and provided preliminary evidence of its discriminative value in differentiating AD and aMCI. By leveraging deep learning techniques to enhance the efficiency and reliability of EPVS quantification, our findings highlight the potential utility of glymphatic imaging markers in the early identification of AD. This approach may contribute to clinical decision-making in the diagnosis and management of cognitive impairment and further facilitate the translational application of glymphatic biomarkers.

## Data Availability

The raw data supporting the conclusions of this article will be made available by the authors, without undue reservation.

## Footnotes

1
https://github.com/simonsf/RTP-Net
