# Supplementary material for: Quantitative assessment of brain glymphatic imaging features using deep learning-based EPVS segmentation and DTI-ALPS analysis in Alzheimer’s disease
Source: Front Aging Neurosci. 2025 Jul 16;17:1621106. doi: 10.3389/fnagi.2025.1621106 (PMC12307369; doi:10.3389/fnagi.2025.1621106)
Supplement: Supplementary file 1 [file Table_1.docx]

**Supplementary Material 1: Post hoc Power Analysis for metrics**

To evaluate the statistical power of the primary group comparisons despite unequal sample sizes, we conducted post hoc power analyses using G*Power 3.1.9.7. As the EPVS-related metrics exhibited non-normal distributions, conventional post hoc power analyses could not be appropriately applied. Therefore, power analyses were performed only for the ALPS indices, which followed normal distributions and were analyzed using one-way ANOVA.

Each analysis was configured using the following parameters:

- Test family: F tests

- Statistical test: ANOVA: Fixed effects, omnibus, one-way

- Type of power analysis: Post hoc – Compute achieved power

- α error probability: 0.05

- Number of groups: 3

- Total sample size: 145 (AD: 89; aMCI: 24; NC: 32)

- Degrees of freedom (numerator): 2

The input effect sizes (Cohen’s f) were derived from observed between-group differences

in means and standard deviations, using the pooled standard deviation across groups.

The computed results are summarized in the table S1.

| **Metric** | **Effect Size (f)** | **Noncentrality Parameter (λ)** | **Critical F** | **Power (1–β)** |
| --- | --- | --- | --- | --- |
| ALPS index | 4.662 | 3151.465 | 3.06 | 1.000 |
| ALPS index-L | 4.042 | 2368.976 | 3.06 | 1.000 |
| ALPS index-R | 4.242 | 2609.212 | 3.06 | 1.000 |
